# Biomimetic scaffolds based on stem cells for neural tissue engineering: a review

**DOI:** 10.1186/s13287-026-05182-3

**Published:** 2026-07-15

**Authors:** Somayeh Kakehbaraei, Cyrus Jalili, Amin Bahreini, Touraj Zamir Nasta, Seyran Kakebaraei, Morteza Arab-Zozani

**Affiliations:** 1 Department of Medical Nanotechnology, Faculty of Advanced Medical Sciences, Tabriz University of Medical Sciences, Tabriz, Iran; 2 Student Research Committee, Tabriz University of Medical Sciences, Tabriz, Iran; 3 Medical Biology Research Center, Health Technology Institute, Kermanshah University of Medical Sciences, Kermanshah, Iran; 4 Neurosciences Research Center (NSRC), Imam Reza General Hospital, Tabriz University of Medical Sciences, Tabriz, Iran; 5 Department of Anatomical Sciences, Kermanshah University of Medical Sciences, Kermanshah, Iran; 6 Social Determinants of Health Research Center, Birjand University of Medical Sciences, Birjand, Iran; 7 Anatomy Department, Kermanshah University of Medical Sciences, Kermanshah, Iran

**Keywords:** Neural tissue engineering, Neural regeneration, Biomaterial, Topographic scaffold

## Abstract

Implementing biomaterials, scaffolds, and stem cell therapy for neural tissue regeneration introduces a revolutionary strategy in regenerative healthcare. By embedding stem cells within intricately engineered scaffolds that replicate the natural extracellular matrix (ECM), remarkable advancements in patient well-being can be realized. These biomimetic scaffolds not only emulate native tissues; they also possess a dynamic, multidimensional structure. Their biocompatibility and capacity to influence cellular metabolism position them as exceptional platforms for bioengineering. The outstanding flexibility of this technique enables optimal selection of biomaterials, scaffold designs, cells, and inorganic materials. A formidable body of evidence is emerging to highlight the vast potential of biomimetic scaffolds in tissue engineering and personalized medicine. Recent scientific studies reveal a significant rise in in vivo testing of biomimetic scaffold-based products, highlighting the critical importance of this research domain and the pressing necessity for continued exploration to facilitate the safe advancement of human-compatible biomimetic tissues and organs. This comprehensive analysis illuminates the vital conditions, challenges, and exciting innovations in scaffold design, especially in the context of brain tissue engineering. Ultimately, this review formulates a comprehensive framework for generating scaffolds that incorporate biomimetic attributes and optimal structures. The innovation of brain-implantable scaffolds holds great promise for reducing the impact of neurological disorders (ND).

## Introduction

**Fig. 1:**
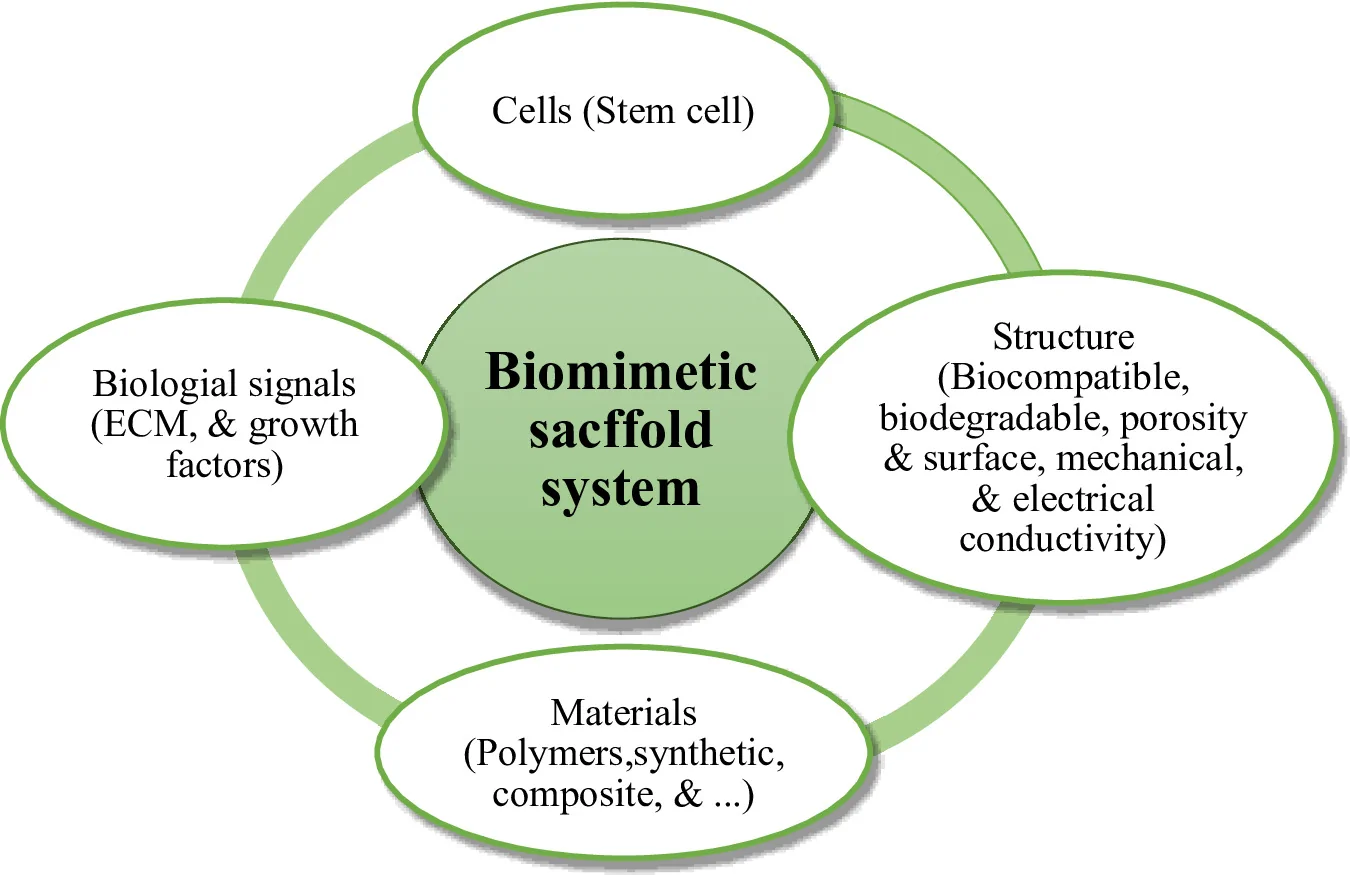
Illustrative overview of a stem cell-derived biomimetic scaffold system

Neurological disorders (ND) are among the leading causes of disability in the human population. As our demographics shift towards an older age group, the prevalence of ND is expected to rise. Traditional approaches, encompassing both drug therapies and surgical procedures, emphasize symptom relief and palliative support instead of targeting the fundamental causes of the condition. There are two main challenges in managing ND: (I) the insufficient structural support required to regenerate damaged neural tissue, and (II) the significant variability observed in individual patients. For instance, people who have experienced a stroke show considerable differences in both neurological changes and behavioral reactions, making it challenging to develop effective neurorehabilitation strategies. Therefore, there is a pressing need to develop new approaches that prioritize personalized medicine [1, 2]. Stem cell-derived biomaterials, celebrated for their self-renewal and differentiation capabilities, are at the forefront of innovative treatments for degenerative diseases, aging, and other complex health conditions. Their remarkable potential lies in their ability to differentiate, ease harvest, have low immunogenic responses, recruit additional host cells, and secrete vital growth factors and matrix proteins, positioning them as a highly promising therapeutic option. While significant technological progress has been made, there remains a pressing need for novel strategies to advance tissue engineering and expand its clinical applications. Within this realm, the foundational triad of tissue engineering comprises scaffolds, cells, and signaling biomolecules (growth factors). The extracellular matrix (ECM) is primarily composed of collagen biopolymers, which exist mainly as fibers and fibrils. A key goal in designing porous scaffolds for tissue engineering is to create a structure from absorbable polymers that closely resembles the ECM, thereby encouraging cell proliferation and organization. Other polymer configurations, such as gels, foams, and membranes, have also been utilized for tissue engineering scaffolds. These scaffolds can be enhanced with signaling biomolecules, whether they are incorporated into the material or bound to the surface [3–5]. Such developmental cues provided by the scaffolds can stimulate cells to secrete extracellular matrix components and facilitate tissue or organ regeneration. The sophisticated tissue engineering scaffold platform shown in Fig. 1 comprises several key components: (1) collections of stem cells that serve as the cornerstone for regeneration, (2) biomimetic materials that provide a variety of biochemical and biophysical signals, mimicking the body’s ECM, (3) engineered frameworks specifically designed to facilitate stem cell migration, and (4) scaffolds’ ability to deliver precise biological signals, including growth factors and cytokines, systematically to influence cellular responses. Tissue engineering is an advanced strategy that integrates biological components with synthetic materials to aid in repairing injured tissues. Specifically, neural tissue engineering focuses on developing methods that mitigate inflammation and scarring often associated with foreign materials, thereby providing a nurturing environment for cellular growth. These scaffolds are crucial for accurately replicating the natural ECM, thereby forming a biomimetic framework that facilitates neural development and growth [6]. An optimal scaffold for neural tissue engineering must possess several crucial characteristics: (I) non-toxicity being paramount, (II) the ability to support neuron survival, proliferation, and migration, (III) enhancement of electrochemical communication between cells, (IV) mechanical properties similar to brain tissue, and (V) the capability to facilitate the controlled release of bioactive substances [1, 7]. Various scaffolds have been effectively developed using a range of materials and methodologies. Among all options, hydrogels show remarkable potential due to their high-water content, which effectively mimics the nature of soft tissues. The distinctive attributes of polyelectrolyte-based hydrogels lie in their ability to control the release of small, oppositely charged molecules, facilitate cargo discharge, and transform their internal configurations in response to external stimuli, including temperature changes, pH alterations, ultrasound exposure, and changes in glucose or urea concentrations [8]. Renowned for its outstanding properties, hyaluronic acid is a natural polymer that has been the subject of significant research due to its ability to promote the survival of neurons and stem cells. This has made it a pivotal element in investigating therapeutic options for ND and different cell replacement therapies [1, 6, 7]. While widely studied, hydrogels exhibit significant limitations, including inadequate mechanical properties, surface roughness, and high impedance, all of which are critical for optimal neuronal development. Consequently, various approaches have been investigated to enhance the mechanical properties of hydrogels, including the incorporation of carbon nanotubes and graphene [9]. Advances in hydrogel conductivity have greatly enhanced neuronal electrical stimulation. However, numerous studies have revealed the toxic effects of carbon derivatives, which can lead to epigenetic changes, chronic respiratory conditions, developmental issues, and other adverse effects. Research into conductive polymers has also been conducted, yet their safety profiles still need to be verified [9, 10]. The limitations in therapeutic approaches for central and peripheral nervous system (CNS/PNS) injuries have catalyzed substantial research into neural tissue engineering techniques to facilitate tissue regeneration. Recent innovations in treating CNS/PNS injuries have involved the application of various biomaterials. This review provides an overview of multiple methods, biomaterials, and the challenges encountered in creating biomimetic scaffolds, while highlighting the critical recent advancements in neural tissue engineering and their impact on improving neural function.

## Landscape: source of neural stem cells, characteristics, and applications

Neural stem cells (NSCs) play a pivotal role in facilitating plasticity, learning, and memory within the adult nervous system. Additionally, they are considered a promising avenue for transplantation therapies to combat neurodegenerative diseases. The adult mammalian brain contains these cells in two essential locations: the subventricular zone of the lateral ventricles and the subgranular zone of the hippocampal dentate gyrus [11, 12]. Notably, hippocampal neural stem cells are essential for learning, memory formation, behavioral regulation, and disease pathology, underscoring the need for deeper insights into their behavior [13]. Recently, biophysical and mechanical factors (such as stiffness, viscoelasticity, spatial confinement, and curvature) have been recognized as significant yet relatively under-researched influences on NSCs. Initial in vitro studies demonstrated that softer two-dimensional substrates, which resemble the stiffness of brain tissue, promote neuronal differentiation of NSCs, whereas stiffer substrates hinder neurogenesis [14, 15]. This finding is particularly significant given the variations in tissue stiffness found within the hippocampus. The mechanisms driving this behavior are beginning to be understood, but further research is essential to determine whether NSCs exhibit similar mechanosensitivity in 3D and in vivo environments. In addition to stiffness, NSCs encounter a range of mechanical inputs during processes such as development, injury, and disease. It is crucial to explore the effects of these inputs to gain a deeper insight into how biophysical cues influence NSCs. Although prior research has clearly demonstrated that NSC proliferation and differentiation are responsive to mechanical factors, the existing experimental frameworks fail to comprehensively capture the ongoing, dynamic influence of stiffness on NSC behavior [16]. NSCs can be derived from a multitude of sources, including skin [17], embryonic stem cells (ESCs) [18], embryonic NSCs [19], bone marrow [20], adipose-derived mesenchymal stem cells (MSCs) [21], induced pluripotent stem cells (iPSCs) [22], and both fetal and adult nervous systems [23–25]. These diverse origins have been instrumental in demonstrating their potential to combat various neurodegenerative disorders by producing specific types of central nervous system cells; for example, mesenchymal stem cells (MSCs) obtained from various sources have been used to generate dopamine-producing neurons to treat Parkinson’s disease [26]. Similarly, numerous other cell types are being harnessed to combat neurodegenerative disorders [27, 28] and to aid recovery from stroke [29]. NSCs represent an invaluable resource for applications in tissue engineering, regenerative medicine, disease modeling, and drug screening. Like other stem cell types, NSCs are strongly influenced by their microenvironment. Previous research using NSCs as a model system, in conjunction with engineered biomaterials, has yielded critical insights into how biophysical factors govern stem cell proliferation, differentiation, and maturation. A wide array of stem cell sources has been investigated to enhance spontaneous recovery from most CNS injuries, yet they often yield limited selections of cell phenotypes. Recently, induced pluripotent stem cells (iPS) have appeared as a viable option for autologous transplantation. Nonetheless, a key disadvantage of these genetically altered cells is their elevated cancer risk, which mainly stems from the integration and recombination of retroviral vectors. Consequently, embryonic stem cells (ESCs) and adult somatic stem cells, especially NSCs, are currently the most viable candidates for clinical neurological applications. NSCs, in particular, are considered the most promising cell type for cell-mediated therapies in neural disorders because they originate from the same tissue as the damaged cells, rendering them exceptionally capable of replenishing injured tissues and responsive to local environmental signals that direct their differentiation [30–32]. Today, the idea of using NSCs as a boundless reservoir of neurons and glial cells for cell replacement therapies targeting brain and spinal cord disorders is no longer just a distant aspiration but a tangible objective. ESCs, as well as stem cells derived from fetal and adult CNS or other tissues, are promising candidates for cell replacement therapies and can differentiate into various adult CNS cell types [33, 34]. Remarkably, researchers have achieved successful rejuvenation of brain function in animal models by using NSC transplants [35–37]. This emphasizes a regulatory mechanism that governs stem cell biology in the adult brain. Given the significant potential of NSCs to treat various debilitating hereditary and acquired NDs, extensive molecular profiling studies have been conducted to identify new markers and regulatory pathways.

## Neural stem cells (NSCs) and interactions with biomaterials

Mechanotransduction refers to how cells respond to mechanical stimuli via pathways regulated by specific transcription factors. The substrate topography and ligand presentation significantly impact these processes. This is especially crucial during neurogenesis when it is vital to rearrange the cytoskeleton and interact with the extracellular environment. Integrins play a key role in attaching to the ECM, enabling bidirectional signaling, initiating direct mechanotransduction, and affecting gene expression, repair, regeneration, growth, and differentiation [38, 39]. Consequently, biomaterial substrates can be designed with tailored nanoscale characteristics to influence the specialized behaviors of NSC, enhancing our understanding of neural development and the implications of various diseases. Given that cell-ECM interactions vary significantly between 2-dimensional (2D) and 3D systems, it is essential to comprehend their effects—particularly in three-dimensional models—on both normal and pathological responses. This understanding is vital for advancing our knowledge of healthy neural tissue and translating these insights into therapeutic strategies for neural disorders [40, 41]. The enhancement of biomimetic and biomechanical properties has been significantly observed through the cultivation of NSCs on electrospun 2D and 3D constructs composed of specifically cross-linked, functionalized self-assembling peptides (SAPs). An in-depth examination of the morphology, architecture, and secondary structures of the electrospun scaffolds—analyzed in both solid-state and within the electrospinning solution—was thoroughly performed. Findings from the morphological investigations indicate that the inclusion of mixed peptides and surfactants markedly improves fiber quality, yielding thinner, more consistent, and flawless structures. In addition, the continuous identification of beta-sheet conformation—a critical signifier of self-assembly—has been consistently noted across the entire process, except for the phase involving the electrospinning solution. The experimental results indicated that NSCs placed on electrospun SAP scaffolds in both 2D and 3D conditions showed exceptional proliferation, viability, and differentiation, significantly surpassing conventional benchmarks. Additionally, the in vivo evaluation of biocompatibility confirmed the successful acceptance of the implanted fibrous channels, reflected by a manageable foreign body response [42, 43]. The basement membrane of the ECM is recognized as a dynamic and adaptable structure that plays a crucial role in regulating cellular activities. Its physiological architecture features a hierarchical arrangement of nanofibers, highlighting the importance of substrate topography. Given this context, numerous studies have focused on utilizing electrospun materials to replicate the in vivo nanofibrous architecture, demonstrating that physical structures have a significant influence on cell behavior [44]. For instance, the fiber diameter in laminin-coated electrospun polyethersulfone (PES) mesh dramatically affects the differentiation and proliferation of NSCs. This research used a systematic approach with various electrospun nanofibrous scaffolds, demonstrating that changes in fiber diameter effectively modulate cell behavior. As a result, different scaffolds led to distinct patterns of lineage-specific differentiation and proliferation in NSCs. Notably, an increase in fiber diameter correlated with a decrease in cell proliferation, indicating that the rearrangement of the cellular cytoskeleton is critical in regulating and enhancing cellular proliferation [45]. The orientation of polycaprolactone (PCL) nanofibers plays a crucial role in determining the morphology, proliferation, and neural differentiation potential of both embryonic and adult NSCs. This phenomenon elucidates how topographical characteristics can modulate stem cell differentiation. Research indicates mature NSCs exhibit distinct responses when cultured on randomly aligned fibrous substrates. Specifically, the cultivation of mature NSCs on aligned fibers markedly enhances their propensity to differentiate into neural lineages compared to those on random fibers. Additionally, the fibers’ size significantly impacts neuronal differentiation, with optimal differentiation occurring at a fiber diameter of 480 nm. These observations imply that the interplay between fiber topography and cell lineage specification influences cellular alignment by modifying cell-substrate interactions, subsequently triggering specific intracellular signaling pathways [46, 47]. Moreover, the topographical characteristics of polyphenylene sulfone (PPSu) scaffolds—particularly those made of random or aligned electrospun nanofibers—significantly affect the behavior of NSCs [48]. Furthermore, recent studies have shown that electrospun PCL fiber mat scaffolds, designed with varying diameters to mimic anatomical features seen during neural development—specifically, fibers resembling radial glia (approximately 0.5-5 microns in diameter) and those similar to small vessels (approximately 10 microns in diameter)—elicit distinct migratory and morphological responses from NSCs [44]. Topographical features may influence various migration phenotypes and lead to morphological alterations. To summarize, the physical attributes of the stem cell niche microenvironment are essential in controlling stem cell fate. Subsequent investigations should focus on elucidating the intermediary processes that connect intracellular alterations and signaling pathways with external topographical cues. Gaining insight into these mechanisms will enhance our understanding of stem cell biology and improve our ability to manipulate stem cells for therapeutic applications.

### Biomaterials: regulating the fate of NSCs

Scaffold biomaterials include various natural, synthetic, and composite materials, complicating their classification. The development of these biomaterials and the release of bioactive molecules, like neurotrophins, play a crucial role in determining NSC differentiation. Given that differentiation is associated with cell survival, it is tough to untangle the effects of these factors on NSC differentiation from their effects on proliferation and survival. Researchers have investigated various strategies to enhance NSC progeny beyond biochemical signals. These include preconditioning NSC populations with supportive cells that facilitate neurogenesis, selectively isolating cells for specific progenitor or precursor types, and employing pharmaceuticals that promote microtubule stability [49, 50]. Research shows that Hyaluronic acid hydrogels infused with higher concentrations of bone morphogenetic protein 4 (BMP-4) significantly enhance astroglial differentiation and boost the survival rates of transplanted cells. Conversely, environments with lower BMP-4 levels and higher Brain-derived neurotrophic factor (BDNF) concentrations show reduced cell survival, despite increased neuronal differentiation among surviving cells. This presents a fascinating inverse correlation between cell survival rates and overall tissue repopulation. This phenomenon may serve as a model to elucidate the selective removal of certain neurons during brain development. During this pivotal phase, neurons are prone to apoptosis while simultaneously extending their axons. Interestingly, the caspases that initiate cell death may also unexpectedly generate mitogens that encourage the proliferation of NSCs. The integration of neural progenitor delivery with spinal interneurons presents a compelling approach to overcoming difficulties in cell survival and differentiation [51]. Soft hydrogel scaffolds in nervous system research adeptly replicate the biochemical and mechanical dynamics of the ECM, ultimately influencing cellular functions [52]. The integration of arginine-glycine-aspartic acid (RGD) sequences into scaffolds made of acrylamide and PEG is a prime example of how these materials can significantly influence NSC behavior with respect to cell attachment, self-renewal, and differentiation. In addition, agarose scaffolds infused with platelet-derived growth factor (PDGF)-AA have greatly facilitated the conversion of NSCs into oligodendrocytes [53, 54]. Research findings show that administering interleukin-6 (IL-6) to neural precursors 6–7 h after transient middle cerebral artery occlusion not only protects against cell death but also induces vascular endothelial growth factor (VEGF) release, thereby promoting angiogenesis in the targeted region. Hydrogel scaffolds made with RADA 16 − 1 IKVAV (isoleucine-lysine-valine-alanine-valine) have effectively served as guiding cues that promote NSC adhesion and differentiation in both in vitro and in vivo environments, while also enhancing neuron communication through their electrical conductivity [55].

In addition, a research aimed to explore the influence of cyclic uniaxial strain on neural crest stem cells (NCSCs) and to assess biomaterial moduli, with the goal of understanding how vascular mechanical strain affects their differentiation into smooth muscle lineages. The application of mechanical strain not only enhanced NCSC proliferation but also promoted their transformation into smooth muscle cells while inhibiting their development into Schwann cells [56, 57]. Additionally, sinusoidal inertial forces were applied to cultured neural stem cells at frequencies of 12.5 Hz, 25 Hz, and 50 Hz, with acceleration amplitudes of 0.25 G or 0.5 G, which could influence their behavior. Notably, Mechanical vibrations at 25 Hz were most effective at promoting cell proliferation at a force of 0.25 G, possibly by reducing apoptosis. Furthermore, NSC differentiation appears to be affected by acceleration amplitude, suggesting that mechanical vibrations may help maintain specific stem cell properties [58]. The interplay between biomaterials and NSCs can be viewed as a bridge linking physical and chemical material properties to the biochemical regulation of neural cells, driven largely by the materials themselves. However, the impact of NSCs on biomaterials, especially in vivo following transplantation, has received scant attention. Only a limited number of studies have addressed the degradation, integration, and erosion of biomaterials or scaffolds surrounding neural tissues after their implantation in the brain. Regrettably, there is a shortage of literature on how NSCs or tissues influence the properties of materials and scaffolds, which are crucial in clinical applications. Conversely, extensive research has focused on understanding the behavior and fate of NSCs, which are governed by materials. Key factors include cell adhesion, proliferation, and differentiation, all of which can be modulated by the biomaterials themselves and by their physical properties and mechanical signals [59–61].

## Application of scaffolds in advancing neural tissue engineering

Effective regeneration strategies for both the CNS/PNS have proven difficult to establish, primarily because our understanding of nerve injury and repair mechanisms remains limited. Studies indicate that, to bridge long gaps (> 6 mm in mice, > 15 mm in rats, and longer distances in larger animals), the regenerating proximal axon branches of a disrupted nerve require guidance. Conventional neurosurgical treatments use autologous nerve grafts to fulfill this task [62]. Consequently, regenerating nerves over longer gaps is a complex medical challenge; even minor PNS injuries typically heal by forming a fibrin cable across the gap. This healing process allows Schwann cells to migrate from the nerve terminals, helping organize the Bungner bands and subsequently promoting neurite outgrowth [63]. Following injury, the complex interplay between protein synthesis and degradation in axons is fundamental to the emergence of growth cones. It is crucial to note the distinct differences in myelination processes between the CNS and the PNS. The contributions of astrocytes and oligodendrocytes in the CNS reveal a vital contrast in how these two systems respond to injury [64, 65]. The CNS environment is inherently inhibitory, which significantly limits spontaneous regeneration. This condition is characterized by glial scars and myelin-associated inhibitors, such as chondroitin sulfate proteoglycans [66–68]. Innovative approaches have been employed to improve neurons’ regenerative abilities, integrating cell therapy, biological factors, drug delivery, biomaterials, and tissue engineering. The efficacy of these approaches in regaining functionality varies substantially.

Biomaterials possess outstanding biocompatibility, conductivity, moldability, and mechanical attributes. Such traits make scaffolds highly effective as cell carriers, drug-delivery systems, and facilitators of neuronal and axonal growth. Additionally, they enhance neural regeneration, combat scar formation, and protect the blood-spinal cord barrier (BSCB), leading to their rising prominence in nervous system therapies [69]. Maskanati et al. demonstrated the combined benefits of phycocyanin and hydroxyapatite in promoting NSC differentiation. They created unique chitosan scaffolds infused with phycocyanin and iron-phycocyanin complexes, which are converted into hydroxyapatite (HA) via freeze-drying. Notably, their magnetic evaluation showed that the resulting powders exhibited a saturation magnetization (Ms) near 0.25 emu/g and a coercivity of around 50 Oe (x-axis represents the applied field). Furthermore, the MTT assay results highlighted the superior efficacy of this newly formulated composite scaffold, as cells on the scaffold exhibited neuron-like characteristics (spindle-shaped morphology with cytoplasmic extensions). These findings point to neuroinductive properties, suggesting both cytocompatibility and promising neuroregenerative potential, positioning it as a strong candidate for neural tissue engineering projects [70]. A groundbreaking study examined the effects of an innovative nanoscaffold infused with stromal cell-derived factor 1 (SDF-1) on NSC activity and synapse formation after traumatic brain injury. The study developed a new nanoscaffold that achieves a Young’s modulus of approximately 3.21 kPa, closely resembling neural tissue mechanics. This innovative technology markedly improves initial cell adhesion, promotes neurite outgrowth, and enhances NSC migration and differentiation, which are vital for neural regeneration in vitro. Furthermore, it enhances NSC survival and integration, promoting synaptogenesis and neuronal regeneration while mitigating neuroinflammation and supporting functional recovery. These discoveries indicate that this groundbreaking Nano-SDF could offer a revolutionary approach to treating traumatic brain injuries by optimizing NSC performance and fostering synapse formation [71]. Wang et al. have introduced a revolutionary composite scaffold integrating 3D graphene foam (3D-GF), GelMA-PEG hydrogel, and small extracellular vesicles (sEVs) sourced from mesenchymal stem cells. This cutting-edge design effectively replicates the brain’s mechanical and electrical microenvironment while delivering vital bioactive factors. The outcome is a synergistic network that provides mechanical support, facilitates electrical signaling, and regulates molecular activity, closely resembling the brain’s natural in vitro conditions. The scaffold’s 3D-GF component exhibits outstanding electrical conductivity and biocompatibility, enabling in vitro development of a brain-like endogenous neural network. The combination of the composite hydrogel with 3D-GF further optimizes the scaffold’s compatibility with the brain’s physical attributes. Results from in vitro experiments demonstrate that NSCs in these composite scaffolds proliferate approximately 7-fold faster than in the control group and achieve a 3-fold higher differentiation efficiency. By facilitating mechanical support, electrical signal transmission, and molecular regulation, this scaffold successfully creates a biomimetic microenvironment. This multimodal approach transcends traditional limitations and offers a promising biomimetic strategy for repairing degenerative diseases of the central nervous system [72]. In stem cell delivery and transplantation, biomaterial paradigms have proven exceptionally effective. By incorporating implantable biomaterial-based scaffolds, we can significantly enhance cell survival, as they successfully replicate the intricate in vivo cellular microenvironments [73, 74]. The delivery of stem cells with scaffolds has shown significant improvements in tissue structure, cell retention, and integration with host tissues, likely due to enhanced cell migration [75]. In contrast, environmental factors can profoundly affect cell fate and function by influencing how cells interact with scaffolds, emphasizing the vital importance of scaffold design in guiding cell behavior and morphology. A new investigation has highlighted that NSCs, when placed on electrospun self-assembling peptide (SAP) scaffolds in both two and three-dimensional environments, showed improved proliferation, viability, and differentiation compared with established benchmarks. An in vivo biocompatibility assessment further reinforced the acceptability of implanted fibrous channels, as evidenced by a manageable foreign body response. The research demonstrates that fibrous electrospun SAP scaffolds (2D/3D), which evoke a suitable foreign body response after implantation in fibrous channels of the spinal cords in rats, indicate the promising potential of custom-designed SAP microchannels as fibrous neural conduits that mimic natural function for spinal cord injury (SCI) regeneration [43].

### Application of scaffolds in spinal cord injury

Scaffolds serve as vital biomaterials in SCI. Typically, these materials are implanted surgically into lesions and synthesized via electrospinning, thereby reducing reliance on organic solvents. In the spinal cord, axons tend to arrange themselves longitudinally, and electrospinning can fabricate scaffolds that accurately mimic this natural arrangement. The most prevalent materials used in SCI scaffolds are chitosan, gelatin, PCL, and poly(D, L-lactide-co-glycolide) acid (PLGA), all of which support axon regeneration, exhibit anti-inflammatory effects, and promote effective tissue repair [76, 77]. A study by Du et al. compared the effectiveness of gelatin sponges and PLGA scaffolds and found that gelatin scaffolds may offer superior results for SCI treatment. It appears that gelatin scaffolds could be a more advantageous option for transplantation, facilitating recovery following SCI [78]. While current interventions for SCI are in place, effective strategies to promote axonal outgrowth and restore function remain elusive. The primary barriers to successful SCI repair include the formation of an inhibitory microenvironment, glial cell proliferation, and demyelination at the injury site—factors that significantly impede axonal regeneration. A synergistic approach that integrates multiple techniques has led to innovative repair solutions, including the use of biomaterial scaffolds as a supportive substrate for axonal growth, modulation of neurotoxicity and immune responses, and application of neurotrophic growth factors to mitigate glial scarring [79, 80].

Ma et al. successfully utilized collagen sponges as carriers for NSCs and infused them with basic fibroblast growth factor (bFGF) to promote NSC proliferation. Their findings showed a notable increase in proliferation, introducing a groundbreaking, efficient, natural biological neural scaffold that could directly influence and possibly create synergistic effects on the regeneration of the nervous system [81]. Zhang et al. utilized electrospun collagen nanofibers to culture rat astrocytes and dorsal root ganglia (DRGs). The study revealed that both astrocytes and DRG neurites aligned along the nanofiber axes, effectively responding to topographical guidance in SCI repair. A hemi-sectioned SCI rat model was employed to assess the reparative effects of electrospun collagen microfibers, demonstrating significant neural fiber infiltration toward the scaffold and a considerable reduction in acute inflammatory responses [82]. Rey et al. utilized 3D Nichoid scaffolds to create a microenvironment similar to the ECM, which supports the proliferation and differentiation of stem cells for neural tissue engineering [83]. Following the implantation of these Nichoid scaffolds in a mouse model of traumatic SCI, the researchers analyzed the changes in gene expression of neural precursor stem cells (NPCs) by evaluating the biochemical signals produced in response to the mechanical stimuli of the scaffolds [84, 85]. The findings of this study established that the PI3K-Akt-mTOR, focal adhesion, and integrin-mediated cell adhesion pathways are key components of NPC adhesion and cytoskeletal development. In addition, the Nichoid scaffold facilitated NPC proliferation via the activation of the p53 and Wnt signaling pathways. This research also revealed the crucial roles of MAPKs, Ras-associated protein-1 (Rap1), Forkhead box O (FOXO), and the Hippo signaling pathways in nerve regeneration and repair following SCI, with significant implications for enhancing stem cell therapy in regenerative medicine. Furthermore, a methacrylate gelatin scaffold coated with graphene oxide was developed to enhance the differentiation of mesenchymal stem cells (MSCs) [86]. These scaffolds can be designed in various forms and may include components that act as templates for cellular proliferation or as stimulants for chemical or physical interactions. The physicochemical characteristics of the scaffold play a crucial role in mediating cellular interactions with the material, thereby influencing the overall success or failure of the implant [87].

### Application of scaffolds in brain regeneration

The brain is the most intricate organ in the human body, composed of a vast array of neurons interconnected by a complex network of neurites. Following an injury, immune activation, coupled with limited innate repair mechanisms, poses significant challenges for regenerating damaged tissue. As a result, existing approaches for brain tissue regeneration remain inadequate. However, recent research has explored the use of biomaterial scaffolds to treat brain injuries, revealing their considerable promise in facilitating recovery. A thorough investigation revealed the development of biodegradable hydrogel scaffolds made from hyaluronic acid, combined with PLGA microspheres infused with vascular endothelial growth factor (VEGF), angiopoietin-1 (Ang1), and an antibody targeting the Nogo receptor (NgR-Ab). These hydrogels were implanted in a mouse model of middle cerebral artery occlusion, demonstrating the scaffolds’ compatibility with brain tissue, their ability to reduce gliosis and inflammation after implantation, and their role in promoting neural regeneration [88]. These advanced hydrogels facilitate neuronal cell migration, maturation, and neurite formation. Meanwhile, Fernández-García et al. have created silk fibroin hydrogels that gel in situ, with their solidification triggered by sonication. These hydrogels can be injected into the mouse brain, and their integration into brain tissue can be precisely modulated by the sonication intensity and duration. Their findings confirm the excellent biocompatibility of these hydrogels within the brain, paving the way for their potential application in traumatic brain injury (TBI) treatment [89]. Recent findings indicate that using collagen glycosaminoglycan scaffolds in TBI-damaged areas yields much poorer results than those observed in the control group. The data demonstrate that when the collagen glycosaminoglycan scaffold is applied in an animal model, it notably facilitates functional recovery and promotes the proliferation and differentiation of endogenous neural precursor cells (NPCs) in both the intra-matrix and perimeter zones of the lesion [90]. Hoban et al. conducted a study on MSCs that overexpress GDNF, which were encapsulated in collagen gel for intra-striatal transplantation. Their findings revealed a notable reduction in microglial cells within the graft zone and an increase in astrocyte recruitment, all while maintaining cell viability and consistent GDNF secretion [91]. Zhong et al. conducted a related study in which they developed a unique biopolymer hydrogel matrix containing cross-linked hyaluronan and heparin sulfate to foster a suitable environment for transplantation into the infarct cavity post-stroke. Their findings indicate that modifying local conditions during stem cell transplantation improves cell survival and reduces stress levels. Employing a pro-survival hydrogel matrix to administer stem cells into the infarct cavity may provide an effective strategy for stroke recovery [92].

**Fig. 2:**
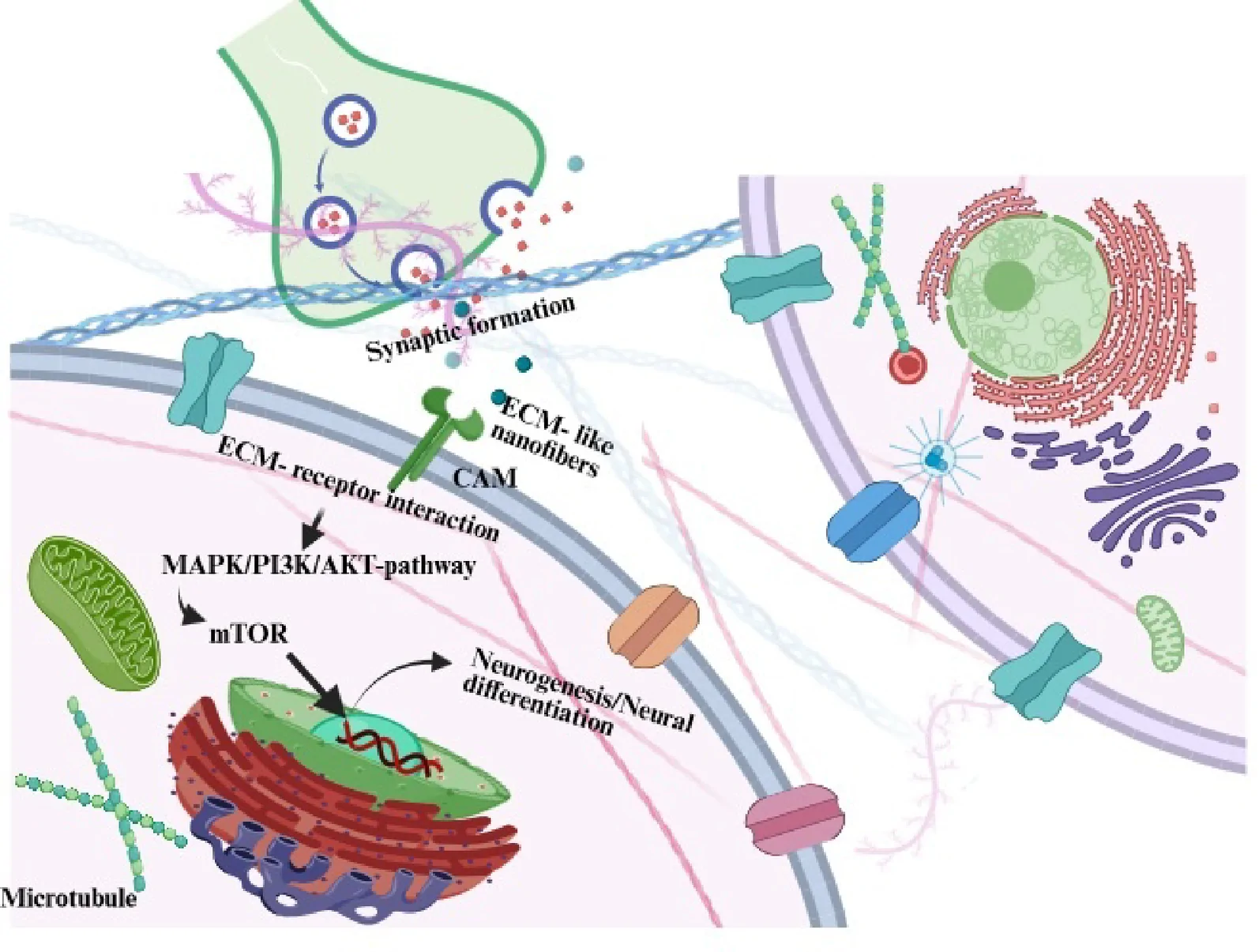
An illustrative diagram showcasing the pathways that could play a role in boosting the neurogenesis of NSCs. The interaction of NSCs with nanofibers that emulate the ECM highlights the importance of growth cones, which consist of microtubules and actin filaments, along with focal adhesions, in regulating cell behavior. These cytoskeletal protein structures are vital for promoting neurite outgrowth (neurogenesis/neural differentiation) on biomimetic scaffolds. Neural growth cones and focal adhesions serve as critical conduits between ECM-like signals from 3D nanostructures and neuronal function, thereby enhancing activation of the CAM-mediated MAPK/PI3K-AKT signaling pathway (Created with www.biorender.com)

Expertly designed scaffolds are aimed at enhancing the regeneration of auditory tissues while supporting tissue healing and the restoration of functionality in affected organs. These components create an optimal spatial framework, guaranteeing cell viability and fostering intricate interactions within the surrounding microenvironment. The effective functioning of mammalian auditory systems is mainly reliant on inner ear hair cells, which transmit auditory stimuli to the cochlear nucleus through spiral ganglion neurons (SGNs) [93]. Therefore, the ongoing development and enhancement of spiral ganglion neuron (SGN) regeneration are vital for effective hearing restoration. A diverse array of scaffolds has been innovatively created to facilitate the recovery or regeneration of auditory function, including Matrigel, electrospun fibers, hydrogels, and those derived from cutting-edge 3D printing technology. In their research, Wille et al. engineered a biodegradable scaffold from polyglycolide and PCL, which was further augmented with a heparan sulfate (HS) coating to enhance neuronal growth [94]. Yan et al. adopted a 3D Matrigel scaffold for the in vitro culture of SGNs. Over six months. Their research highlighted that this 3D culture system considerably promoted neurite growth in SGNs by augmenting the surface area of growth cones and enhancing synaptic density. The scaffold crafted in their study was essential for supporting SGNs, improving their in vitro survival by delivering BDNF, which possesses neuroprotective properties. The findings underscored the synergistic effects of HS and BDNF in inner ear therapy, leading to improved neurite growth in SGNs and facilitating neuronal guidance [95]. Additionally, researchers developed an innovative nanofiber scaffold composed of aligned poly-L-lactic acid (PLLA) and polycaprolactone nanofibers, utilizing a mixing ratio of 4:1. Following a Matrigel coating, these composite nanofibers were surgically implanted into a guinea pig model using a cochlear duct approach. According to Hackelberg et al., aligned composite nanofiber scaffolds demonstrated a pronounced superiority over random scaffolds in fostering neurite growth and steering neuronal differentiation, particularly for the regeneration of the auditory nerve. These findings suggest that despite being an emerging therapeutic avenue with limited research, neural scaffolds hold significant promise for restoring or regenerating auditory function [96]. Also, neural cells cultured on these scaffolds release specific biochemical factors in response to topographical signals, which modulate intracellular signaling pathways and create feedback loops with surrounding host cells. Studies indicate that various signaling pathways, including the Wnt/β-catenin, mitogen-activated protein kinase (MAPK), and phosphatidylinositol 3-kinase (PI3K)/Akt pathways, are crucial in mediating the biological responses of nerve cells in the context of extracellular matrix-like scaffolds [97, 98]. The underlying mechanism by which neurons respond to 3D nanostructures was uncovered through mRNA sequencing. As NSCs adhere to nanofibers that mimic the ECM, growth cones and focal adhesion play a crucial role in modulating cell behavior [99]. At the tip of growing axons lie growth cones, sensitive structures built from microtubules and actin filaments. These components create a dynamic meshwork that equips the growth cones with the ability to detect morphology-related signals [100]. The intricate protein assemblies known as focal adhesions, which connect the cytoskeleton to adhesion substrates through the activation of cell adhesion molecules, are proven to be vital for neurite outgrowth on biomimetic scaffolds. The interactions formed by integrins are critical as they initiate signal transduction by activating calcium signaling, subsequently leading to significant changes in cell morphology and biological functionality [101, 102]. The hypothesis puts forward that neural growth cones and adhesions function as crucial links between the ECM-mimicking signals of 3D nanostructures and the actions of neural cells, promoting the activation of the CAM-mediated MAPK/PI3K-AKT signaling pathway [99] (see Fig. 2). As we move closer to clinical implementation, it is essential to prioritize pharmacokinetic parameters and biodistribution studies in animal models, conduct thorough long-term safety evaluations, and enhance minimally invasive delivery techniques. By thoughtfully combining molecular data, functional outcomes, and biocompatibility standards, we can effectively move the most promising candidates into Phase I-II human trials. While substantial challenges lie ahead, the robust preclinical in vivo evidence presented here provides a vital foundation for developing regenerative therapies for conditions that currently lack effective treatments. Our strategic focus has identified three clinical trials examining the synergy between scaffolds and NSC in treating spinal cord injuries and other nervous system conditions, all listed on ClinicalTrials.gov. NCT02688049 is a Phase II randomized trial involving 30 participants (males and females aged 18–65) with SCI at cervical and thoracic levels (C5-T12). This trial included the surgical implantation of the NeuroRegen Scaffold™, along with local administration of MSCs or NSCs. The primary endpoints focused on safety and systemic inflammatory markers at the 6-month mark, while the secondary endpoints targeted improvement in the American Spinal Injury Association (ASIA) Impairment Scale (AIS grades A–C) and the development of motor-evoked potentials over 24 months. The enrollment process was completed in 2018. The Phase I study NCT02352077, a single-arm trial, enrolled 8 patients (7 men and 1 woman, with a mean age of 31.5 years) with chronic thoracic spinal cord injuries (C5-T12) who received the NeuroRegen Scaffold™ containing stem cells. No adverse events related to the treatment were recorded during the one-year follow-up period. Preliminary findings suggest that integrating the NeuroRegen scaffold with human umbilical cord mesenchymal stem cells (hUCB-MSCs) is a feasible approach for managing SCI.

NCT06361433 (RAINBOW-Hx) is a groundbreaking ongoing Phase I-II open-label study that commenced in 2023. The trial aims to enroll eight participants aged between 20 and 70 years who are suffering from chronic sequelae of intracerebral hemorrhage, focusing on the stereotactic injection of HUFF-01, an innovative autologous bone marrow stromal cell-loaded scaffold. The main endpoint is safety after 12 months, and the study will also evaluate functional outcomes through neurological assessments, magnetic resonance imaging, 18 F-fluorodeoxyglucose positron emission tomography, and 123I-Iomazenil single-photon emission computed tomography, which will be performed for 1 year following administration. Collectively, these three trials will mark the inaugural human applications of electrospun scaffold-stem cell constructs, directly leveraging strong preclinical in vivo data that underscores their translational potential.

## Manufacturing techniques and biomaterials utilized in the design of scaffolds for neural tissue engineering

**Fig. 3:**
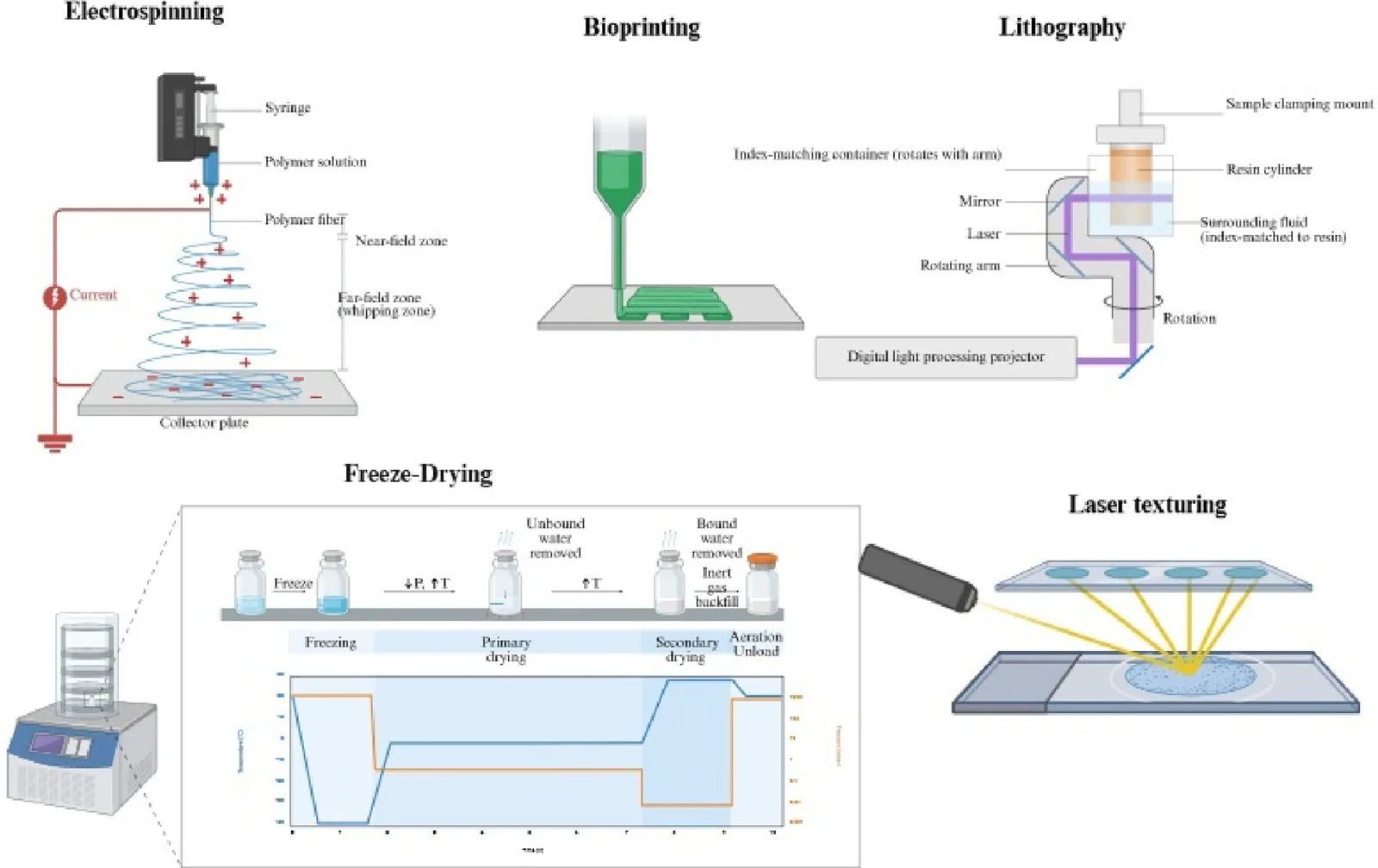
Diverse techniques for the development of biomimetic neural scaffolds. Several state-of-the-art fabrication methods, including electrospinning, bioprinting, lithography, freeze-drying, and laser texturing, have been introduced to generate sophisticated structures from natural and synthetic polymers. The central purpose of these developments is to engineer scaffolds that faithfully imitate the physical, chemical, and mechanical properties characteristic of neural tissue (Created with www.biorender.com)

The evolution of tissue engineering has led to the development of various components, including scaffolds and integrated technologies. Numerous fabrication methods have been established to construct complex structures from natural and synthetic polymers. The primary objective has been to develop scaffolds that mimic the physical, chemical, and mechanical properties of neural tissue. Achieving this requires scaffolds to possess vital characteristics, including pores, fibers, and channels at both the nanoscale and the microscale, which are indispensable for neural tissue engineering applications. The adaptability of biomaterials, whether synthetic or natural, is primarily due to their various forms, such as sponges, conduits, nano/microspheres, hydrogels, and nano/microfibers, which can be specifically designed to address the requirements for repairing the CNS/PNS systems. However, Rapid prototyping (RP), often referred to as solid free-form fabrication, encompasses techniques such as 3D bioprinting, stereolithography, and selective laser sintering. Significant advancements have been made in various processing strategies, including electrospinning, 3D bioprinting, and their combinations. These methodologies enable precise control over the uniformity and physical characteristics of scaffolds, including porosity, hydrophilicity, hydrophobicity, and swelling ratio. Furthermore, they promote favorable interactions between cells and biomaterials within a tailored 3D microenvironment [59, 103]. Initially, the scaffolds were primarily designed for drug delivery, but they have since found significant applications in tissue engineering. Traditional methods permit the design of scaffolds with specific pore sizes and shapes; however, they often fall short of achieving intricate internal architectures and adequate connectivity [104]. The solvent-casting technique entails dissolving a polymer in an appropriate solvent and subsequently adding an insoluble salt. As the solvent evaporates, a salt-polymer composite forms and is then immersed in water, resulting in a highly porous structure as the salt is leached out [105]. A significant advantage of this method is its capacity to produce scaffolds with enhanced porosity and customizable pore sizes. However, it has limitations: it can only generate simple scaffold structures, such as flat sheets and tubes. Additionally, residual solvents can pose toxic risks to cells and tissues [106]. Freeze-drying is a technique in which a polymer is dissolved in a solvent, poured into a mold, and then cooled below its freezing point. This method produces a solidified solvent that sublimates, yielding a resilient scaffold with a complex, interconnected pore structure. Solidification occurs within a temperature range of 20 °C to 80 °C [107]. A major advantage of employing this method for scaffold fabrication is the elimination of high temperatures, which can reduce biological activity or lead to the loss of crucial biological factors integrated into the scaffold [108]. The gas foaming technique is an innovative approach that uses an inert gas as a foaming agent, creating pressure within biodegradable pre-molded polymers immersed in solvents such as water or fluoroform at high temperatures. As the gas applies pressure, it continues until saturation is achieved, facilitating the absorption of gas bubbles into the polymer. This process yields a sponge-like structure with approximately 85% porosity, with pore sizes ranging from 30 μm to 700 μm [109]. Additionally, this approach has been successfully paired with freeze-drying to produce a highly macroporous bone scaffold. The synergy of these methods led to the creation of a distinctive hybrid macroporous bone scaffold featuring 70% interconnected open porosity. Notably, this cutting-edge scaffold exhibited non-toxic properties, and in vitro experiments on human and murine osteoblasts revealed outstanding adhesion and growth [110]. Using a high-voltage electric field, electrospinning is an advanced technique that transforms charged polymer solutions into ultrafine fibers. This innovative process can generate a range of fiber patterns with outstanding porosity. The fibers’ properties, including morphology and diameter, are strongly influenced by key factors such as solution viscosity, polymer molecular weight, charge density, and electric field strength. By skillfully adjusting these parameters, one can fabricate fibers with specific, appealing qualities [111]. Stereolithography, an innovative technique, fabricates solid objects by layering ultraviolet (UV)-curable materials through sequential printing [112]. However, a notable drawback of this method is its reliance on photosensitive resins, which may cause skin irritation and cytotoxic effects. To enhance in vivo biocompatibility, alternative resins sourced from vinyl esters warrant further investigation [113]. Tables 1 and 2 provide a detailed overview of the practical methods and materials used for creating biomimetic neural scaffolds. The table also examines the various polymers commonly used to construct these scaffolds in neural tissue engineering. Consequently, this information helps researchers select appropriate scaffolding approaches to advance brain tissue engineering. (See Tables 1 and 2), (See Fig. 3).

## Application of artificial intelligence in neural tissue engineering

Research activities are actively pursuing the development of innovative techniques and tools to address challenges in tissue engineering, particularly those enhanced by Artificial Intelligence (AI). The intricacies of designing and resolving issues related to tissues and organs pose a significant, resource-intensive challenge. By harnessing machine learning (ML) and deep learning (DL), we can significantly enhance cell culture processes, enabling breakthroughs in organ and tissue engineering [114]. The integration of AI into tissue engineering marks the dawn of an innovative age characterized by greater efficiency and the ability to develop personalized medical devices alongside more effective tissue constructs. Nevertheless, the sector still grapples with significant obstacles, including limited cell availability, challenges in vascularization, immune responses, and insufficient integration with host tissues [115]. AI stands out as a dynamic, interdisciplinary field that significantly impacts the main areas of research. It contributes to (i) computational modeling, where algorithms help evaluate the responses of cells and tissues to various stimuli, leading to a deeper understanding of critical tissue regeneration mechanisms; (ii) biomaterial design, where analysis of extensive datasets allows the prediction of optimal biomaterial composition and structure for targeted tissue engineering applications; (iii) cell culture optimization, leveraging algorithms to identify the best culture conditions for specific cell types by interpreting experimental data; and (iv) personalized medical systems, which enhance therapeutic outcomes and lower the risks of complications by assessing individual patient data, including genetic information and medical history [116, 117]. The fusion of tissue engineering with artificial intelligence offers an exciting opportunity to propel the field into a new era of personalized, effective regenerative therapies. Despite the PNS’s remarkable regenerative capacity compared to the CNS, conventional grafting methods have significant drawbacks, including risks of disease transmission and size mismatches [118]. However, advanced techniques like solvent casting, electrospinning, injection molding, and 3D bioprinting offer groundbreaking solutions for the creation of personalized nerve structures while tackling these issues. Additionally, the vital contribution of ML techniques cannot be overlooked, as they play a pivotal role in enhancing the composition and properties of the biomaterials used to produce peripheral nerve guidance conduits [117]. The study conducted by Koch et al. evaluated the efficacy of nerve guidance conduits by analyzing the nerve gap-to-axon elongation ratio within the graft. By implementing bootstrap-aggregate neural networks, they aimed to predict conduit performance using 40 parameters, grouped into structural, material, form, growth factor, and arrangement categories. Significant findings indicated that printing parameters, including delay time, layer thickness, and orientation, significantly affected the mechanical properties of nerve guidance conduits. The study employed three distinct methods to assess the conduits’ performance, including the predictive capabilities of feedforward neural networks (FFNN) and bagged neural networks (BNN), which were trained using particle swarm optimization (PSO) and swarm intelligence reinforcement learning (SWIRL) [119]. Various tissue engineering strategies have aided in understanding neural development and the brain’s mechanical properties [118, 120]. Concurrently, ML techniques have successfully modeled the brain’s viscoelastic behavior. So far, most ML models addressing hyperelasticity have relied on synthetic data generated by microscopic simulations. In a commendable deviation, Linka et al. have utilized actual experimental data to train neural networks to characterize human brain tissue. They employed ML methods to illuminate the correlation between mechanical properties and the composition of human brain tissue, thereby contributing to the development of biomaterials for neural tissue engineering and 3D printing, where the extracellular components investigated hold potential to improve the biocompatibility and properties of matrix materials [121]. The transformative potential of AI in neuroimaging is nothing short of revolutionary. With exceptional capabilities in analyzing complex imaging datasets, AI plays a crucial role in the swift identification and accurate diagnosis of neurological conditions, including Alzheimer’s disease, brain tumors, and various ND. Its proficiency in analyzing MRI and PET scans not only enhances diagnostic accuracy but also significantly improves patient outcomes [122]. Recent research has highlighted the remarkable capabilities of AI and deep learning algorithms in interpreting intricate neuroimaging data, offering vital insights into these challenging conditions [123]. The oncology field is advancing at an unprecedented pace, largely driven by the use of radiomics and machine learning, which are fundamentally reshaping treatment strategies and the ongoing evaluation of brain tumors [124]. Innovative methodologies now enable the extraction and analysis of quantitative metrics from medical imaging, establishing a robust framework for personalized models that predict disease progression and identify potential therapeutic targets [125]. The incorporation of artificial intelligence significantly enhances the characterization and management of brain tumors, providing healthcare professionals with essential insights that improve decision-making and ultimately lead to better patient outcomes. As research in this field accelerates, the prospects for groundbreaking advancements are ever-expanding.

**Table 1:** Biomaterials and their application in neural tissue engineering

Biomaterial	Pros	Cons	Application	Refs.
Collagen	I. High biocompatibility and Versatility, II. Effective water absorption properties, III. Low cytotoxic and inflammatory, IV. Capability to customize mechanical and cross-linking properties.	Inadequate structural integrity and a lack of mechanical properties in the hydrogel form	I. Axonal regeneration, stem cell therapy	[126–128]
Alginate	I. High biocompatibility and biodegradability, II. Non-antigenicity and Versatility in 3D bioprinting, III. Water solubility. IV. Cytocompatibility	I. Unstable mechanical characteristics, II. Lack of specific cell-recognition sites.	I. Axonal regeneration II. cell-based therapies of neural diseases	[129–132]
Chitosan	I. Biocompatibility, biodegradability, II. non-immunogenicity, III. Drug delivery capability.	I. Potential toxicity, II. Low cellular connectivity.	I. Axonal regeneration, facilitating the release of drugs and neurotrophic factors, and the regeneration of myelin II. Having self-healing abilities and adhering to tissues	[133, 136]
Hyaluronic acid	I. High water levels. II. Low immunogenicity. III. Excellent biocompatibility and viscoelastic properties.	I. non-adherence of cells, II. Restricted mechanical capabilities.	I. Axonal regeneration, Survival of glial cells, neuronal II. delivery of growth factors III. Suitable for cellular transplantation	[1, 130, 137, 138]
Gelatin	I. Excellent biocompatibility and biodegradability, II. Low antigenicity.	Unstable mechanical characteristics, poor electroactive	I.Tissue regeneration, anti-inflammatory properties, survival of cells II. Improving motor function recovery in spinal cord injury	[139–141]
Cellulose	I. Low cost, II. Proper bioactivity, Biocompatibility, III. Nontoxicity. IV. Improving electrically conductive	I. Durability against biodegradation, II. Low resistance to perpendicular compressive distortion, III. Weak porosity.	I.Tissue regeneration, anti-inflammatory properties, release growth factors, axon regeneration, II. Treating traumatic nerve injury	[142–145]
Poly-D, L-lactic-co- glycolic acid (PLGA)	I. non-toxicity, II. Biodegradability. III. Ideal biocompatible	I. Weak bioactivity, II. Long-term strain-induced deformation and failure.	I. Axonal regeneration, vascularization	[146–148]
Poly-L-lactic acid (PLLA)	I. Biodegradable, II. High porosity, III. Enhanced surface-to-volume ratio. IV. Conductivity, antibacterial activity	I. Weak bioactivity and biocompatibility, II. Release of acidic byproducts during the process of degradation.	I. Axonal regeneration, cell survival	[149–151]
PCL	I. Biocompatibility and Biodegradability, II. Good mechanical and elasticity properties, III. Weak toxicity.	I. Weak bioactivity, II. Cytotoxic effects of organic solvent exposure.	I. Axonal regeneration, neurotrophic factors release, stem cell therapy II. Promising alternative to autografts for treating Peripheral nerve injuries	[152–155]
Polypyrrole (PPy)	I. Excellent conductivity, II. Non-toxic, non-mutagenic, and non-allergenic, III. Good biocompatibility. IV. Good conductive, V. long-term structural integrity	I. Non–dissolution, II. non-degradability, III. Weak stability.	I. Axonal repair, drug release, electrical stimulation	[136, 156, 157]
Polyaniline (PANi)	I. High conductivity, II. Biocompatible, III. Antibacterial, IV. Low cost. V. Tensile strength, wettability, degradability	I. Low solubility, II. Poor mechanical, III. Weak cell adhesion, IV. Non-biodegradable.	I. Axonal repair, drug release, electrical stimulation	[158–160]
Carbon nanotubes (CNTs)	I. High electrical conductivity, II. Large surface area, III. High stability. IV. Favorable mechanical properties	I. Cytotoxicity, II. Non-biodegradable.	I. Neurite regrowth, repair of peripheral nerve injuries II. Improving in axonal development, myelination	[161–164]

**Table 2:** Manufacturing techniques in the design of scaffolds and their application in neural tissue engineering

Fabrication	Pros	Cons	Application	Refs.
Electrospinning	I. Effectiveness in fabricating micro-/nanoscale fibrous mesh, II. Effortless to operate, straightforward, highly adaptable, exceptionally efficient, and perfect for large-scale production, III. Structures with a high ratio of surface area to volume IV. High porosity.	I. Restricted regulation of pore structures, II. Poor reproducibility, III. Challenges in designing 3D scaffolds with precise pore structures, IV. Requirement of the high voltage, V. Potential toxicity and utilization of organic solvents.	I. Promoted myelination, axonal growth, and increased nerve Regeneration, II. Fabrication of nerve conduits for PNS regeneration through the sheet rolling process. III. promote motor function, increase tissue remodeling	[165, 166]
Bioprinting	I. Facilitates the creation of both 2D and 3D structures, II. High resolution (50 μm), reliable, and durable III. Integration of cells and macromolecules.	I. Expensive, II. Weak mechanical strength reproducibility, III. Slow processing, IV. Time-consuming, V. A reduced cell density of less than 106 cells/ml.	I. The development of functional neural networks, II. Modeling for nerve regeneration therapies. III. promotes cell adhesion and differentiation	[4, 167, 168]
Gas foaming	I. Creating porous scaffolds, II. Low cost, III. Eliminates the need for organic solvents and high temperatures.	I. Restricted mechanical strength, II. Inadequate pore connectivity, III. Inconsistent pore size regulation.	I. Promoted growth of Schwann cells and peripheral nerve repair, II. The creation of scaffolds, in combination with alternative techniques. III. promoted axon reinnervation and remyelination	[99, 169, 170]
Stereolithography	I. Low cost, II. Good biocompatibility, III. Excellent resolution (25 μm), IV. 3D structural integrity.	I. Expensive specialized equipment, II. The resolution may be diminished due to the diffusion of ink, III. Resins with toxic byproducts.	I. Regeneration of the spinal cord injury II. promising for nerve regeneration through the adherence, proliferation, and survival of Schwann cells	[171–173]
Freeze-drying	I. User-friendly functionality, II. Preservation of bioactivity without an increase in temperature, III. Adjustable pore size is achieved by adjusting the freezing rate and pH levels. IV. Increasing the electrical conductivity and good biocompatibility, strong antibacterial activity	I. Extended processing durations, II. Deteriorated surface structure, III. Complex manufacturing process.	I. Process of regeneration following spinal cord injuries and brain trauma, II. Utilization of nerve grafts in the context of peripheral nerve repair.	[174–176]
Solvent casting	I. Straightforward procedure, II. The dimensions of the pores can be regulated through the use of suitable molds, III. Drugs can be easily integrated into the scaffold structure, IV. Low cost. V. Enhancing mechanical properties	I. Utilization of extremely hazardous solvents, II. Inadequate pore interconnectivity, III. Scaffolds may exhibit residual toxicity, IV. Limited to the fabrication of membranes with a maximum thickness	Development of injectable materials for peripheral nerve injury repair	[177–179]
Laser texturing	I. Negligible impact on adjacent regions, II. Without physical contact, III. Using a variety of material types.	I. Expensive, II. Specialized equipment.	I. The accurate and manageable micropatterning and nanopatterning of scaffolds for facilitating nerve regeneration II. Accelerating the optimal morphological and functional recovery of nerve tissue in the wake of traumatic brain injuries.	[180–182]
Hydrogel formation	I. Adjustable mechanical robustness, II. Compatible and capable of biodegradation, III. Capable of providing regulated drug release, IV. Integration of biological components such as DNA, proteins, and cells. V. Increasing nerve fiber regeneration and inducing the secretion of neuropeptides	I. Certain materials might lack adequate biodegradability, II. The loading of drugs can be problematic, especially for those that are non-hydrophilic, III. There is a risk of toxicity associated with unreacted cross-linking agents.	Creation of scaffolds utilizing bioprinting methodologies and the formulation of bio-inks aimed at facilitating brain delivery and regeneration, improving spinal cord injury	[183–185]

## Fundamental factors in designing scaffolds for neural tissue engineering

**Fig. 4:**
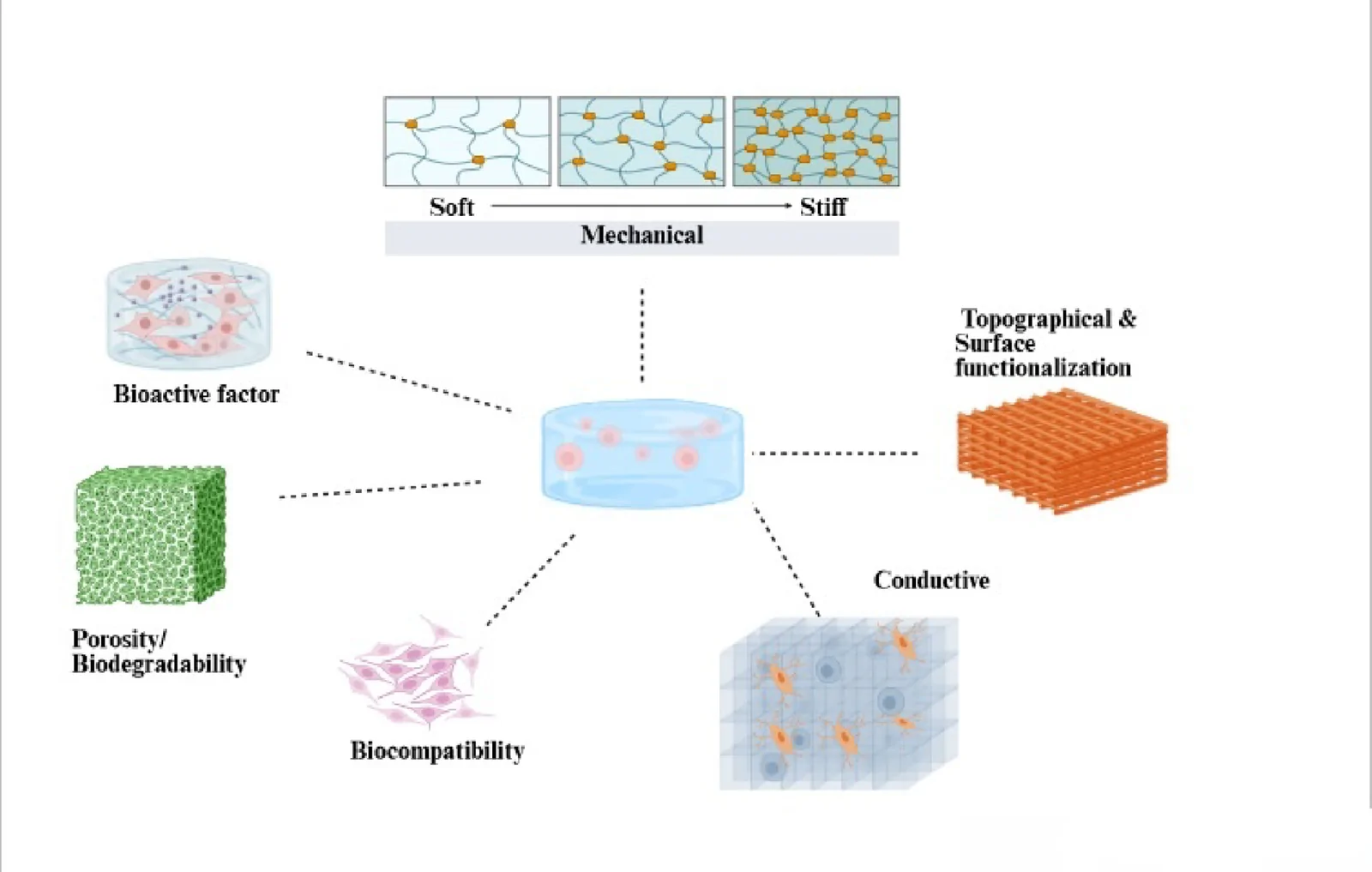
A schematic representation illustrating the essential qualities of the ideal design for scaffolds in neural tissue engineering. Scaffolds, cells, and growth-stimulating factors are the primary components in tissue engineering, and their synergy is vital for producing functional new tissue. An ideal scaffold must meet several key requirements: it should be biocompatible and biodegradable, exhibit suitable mechanical properties, feature an effective structural design, maintain optimal porosity and surface features, demonstrate electrical conductivity, demonstrate bioactivity, and incorporate cutting-edge manufacturing techniques (Created with www.biorender.com)

Scaffolds used in tissue engineering encompass a diverse range of materials and techniques tailored to meet the specific needs of various tissue types. For brain scaffolds, several vital attributes are essential for success. First and foremost, they must be biocompatible and have mechanical properties that match the target tissue’s characteristics. It is essential to assess the ideal surface topography that encourages neuron adhesion, the flexibility to alter surface properties, and the opportunity to integrate a range of substances, including antibiotics, anti-inflammatory agents, growth-promoting factors, and neurotrophic agents. The porosity of scaffolds plays a vital role in facilitating ion exchange and the transport of substances. In this context, they act as a source of nutrition and growth, supporting cell survival and facilitating neural connectivity and regeneration [87, 186] (see Fig. 4).

### The ability of scaffolds to biodegrade and be biocompatible

The primary and most evident attributes of scaffolds utilized in tissue engineering are their biocompatibility and safety. Cell scaffolds must facilitate cell attachment, migration, and proliferation at the cellular level, enabling cells to integrate and form a functional bio-scaffold. Conversely, scaffolds must exhibit non-immunogenic properties at the tissue level to ensure the host body accepts them while avoiding fibrogenic responses. Although numerous materials have been shown to possess biocompatibility within the body, they may still be rejected when located within the brain tissue. Unraveling the complexities of rejection involves a meticulous analysis of the CNS’s unique immunological network, which operates under the protective shield of the blood-brain barrier (BBB)—a fundamental defense against infections and injuries. This immune response, highlighted by an anti-inflammatory M2 state, features anti-inflammatory cytokines that aid in clearing cellular debris, promote ECM repair, and support tissue rejuvenation [37, 38]. Research into the brain’s immune response to cranial cavity-implanted devices has garnered considerable interest. Macrophages respond rapidly following the implantation. These cells stretch their processes to the implant’s surface, reaching around 130 μm. Within just 24 h, the neural electrode becomes enveloped by activated macrophages, leading to the formation of a delicate cellular capsule. This layer may hinder Ionic balances and interfere with neuronal interactions. Within two to three weeks, these astrocytes encase the microglia in a protective sheath. At the four-week post-implantation mark, glial cells form tight junctions, limiting the flow of ions and neurotransmitters. This cascade of events leads to neuronal death and degeneration of neurites, impacting a region of approximately 150 μm [187–189]. Using biodegradable, implantable devices holds great promise for addressing various safety concerns associated with traditional implants. Natural hydrogels have emerged as the most extensively studied materials for creating scaffolds that provide essential mechanical support for neuronal growth and development. However, the toxic effects arising from the degradation by-products of these hydrogels warrant more careful examination in current research. Further studies are needed to understand the long-term effects of these by-products on biological systems [190, 191].

### Mechanical characteristics and porosity

To successfully engineer tissues, scaffolds must exhibit compatibility with the target tissue at cellular and tissue levels. Cells that anchor to surfaces produce contractile forces in their immediate environment and can gauge the rigidity of the materials they interact with. The mechanical properties of hydrogels—such as stiffness, density, composition, orientation, and viscoelasticity—significantly influence how cells behave and their characteristics, affecting morphology, spreading, gene expression, axonal growth, and differentiation. In surgical applications, the mechanical properties of scaffolds must be in harmony with the anatomical site of implantation to reduce friction and provide adequate strength for manipulation [192, 193]. A revolutionary scientific breakthrough has introduced an innovative, non-invasive technique for measuring the elastic modulus of hydrogel, utilizing a customized variant of the Hertz model engineered explicitly for thin films. This strategy greatly enhances the potential to analyze the mechanical characteristics of engineered neural tissues. A comprehensive understanding of the mechanical properties of scaffolds can enhance the design of bioengineered materials intended to promote nerve regeneration after injury [194, 195]. Neurons display numerous activities on both pliable and firm surfaces, encompassing migration, proliferation, neurite extension, and the formation of actin filaments. On the other hand, astrocytes tend to favor proliferation specifically on rigid substrates. The differential growth pattern identified here may offer significant benefits for brain implants, as it enhances neuronal regeneration while potentially reducing glial scar formation [49]. Compared to standard non-porous materials, interconnected porous hydrogels yield remarkable cellular responses. The dimensions of pores are fundamental for the effective diffusion of nutrients and waste materials. An optimal substrate with well-defined pore dimensions boosts cell seeding, enables deeper infiltration, guarantees effective oxygen diffusion, and fosters an even distribution throughout the scaffolds. Moreover, pore size has a significant impact on cell adhesion, intercellular interactions, and cell spreading. Neurons possess the ability to detect the surface of the scaffold. Focal adhesions play a crucial role in managing signaling complexes and integrating functionalities, triggering a signaling cascade that promotes both cell proliferation and differentiation [196, 197]. Extensive research indicates that porosity is essential for fostering a conducive environment for neuronal development. Moreover, scaffolds with intricately designed anisotropic porous structures can be constructed to promote cell proliferation effectively. Studies have identified that the ideal pore size for culturing neurons ranges from approximately 95 to 150 μm [198–200].

### Topography characteristics

The extraordinary polarized morphology of neurons, with their soma and dendrites at one pole and axons at the other, uniquely identifies them among different cell types. When neurites extend in unintended or inappropriate directions, it hinders the effective creation of a functional neural network. Contact guidance, which refers to the movement of cells in response to the topographical features of a surface, plays a vital role in the field of neural regenerative medicine. The physical characteristics of materials, such as their topography, roughness, stiffness, and the presence of fibers or filaments as a 3D scaffold, significantly influence cellular behavior [201]. Current techniques in scaffold development for brain tissue engineering focus on harnessing the natural characteristics of ECM topography. Innovative methods have been employed to create high-resolution surface patterns, facilitating a deeper understanding of cellular interactions with nanoscale topographies [202]. Incorporating nanofibers and aligned carbon nanotubes has been shown to enhance biocompatibility, promote neurite extension, and guide the growth patterns of neurites. Furthermore, the diameter of the fibers plays a significant role in influencing cell differentiation, as evidenced by studies involving neural stem cells cultured on aligned PLLA fibers with a diameter of 250 nm. Although these cells can proliferate regardless of fiber size, it is essential to note that fibers of 250 nm effectively stimulate cell differentiation [203–205]. Within the domain of brain tissue engineering, contemporary strategies for material design must prioritize the topographical properties of scaffolds, which should be specifically designed to create either nano-structured or micro-structured environments. These environments are pivotal for steering cellular growth, controlling cell differentiation, and enhancing the integration of regenerated tissues. Studies have demonstrated that nanostructures can influence axonal extension and lead to distinctive branching patterns. Moreover, the groove or ridge pattern embodies a common type of anisotropic continuous geometry observed in planar topography. Key experimental parameters typically evaluated include the widths and depths of these grooves. Considering the varying sizes of grooves, from a few µm to several tens of micrometers, embryonic hippocampal neurons tend to align parallel to the grooves as their depth increases. Interestingly, these neurons also adapt their polarity in response to changes in topographical characteristics. When the distances between grooves greatly exceed the dimensions of the neural cells, monopolar and bipolar cells can evolve into multipolar cells [206]. The results indicate that differences in material surface properties can significantly influence cellular responses, even in the presence of consistent physical signals and topographical features. The substrate’s chemical and physical properties, together with the surrounding microenvironment and the source of neural cells, play a crucial role in determining cellular behavior. Hence, it is essential to carefully design and control experimental conditions while performing a thorough analysis to identify specific cues and clarify the underlying mechanisms. A summary of the identified topographical patterns and their associated cellular responses is presented in Table 3 (see Table 3).

**Table 3:** Topographical types and their impact on neural cellular function

Biomaterial	Topographical design pattern	Result	Refs.
Tyrosine-isoleucine-glycine-serine-arginine (YIGSR) peptide	Ridge/groove	Increasing Schwann cells proliferation, migration, and elongation, as well as up-regulated gene expression related to proliferation, apoptosis, migration, and axon regeneration	[207]
Chitosan	Ridge/groove	Regulating Schwann cell growth, potential applications in neural regeneration, and facilitating the development of a porous neural conduit featuring a micropatterned inner wall structure	[208]
Chitosan-film	Ridge/groove	Modulates the expression of cytokines, increases the proliferation and migratory abilities of Schwann cells, stimulates neurite extension in cultured motor neurons, and facilitates the regeneration of axons	[209]
Polyurethane acrylate (PUA)	Ridge/groove	Effectively facilitate the differentiation of human embryonic stem cells (hESCs) into neuronal lineages	[210]
Poly(ethylene-vinyl acetate) (EVA)	Grooves	Facilitates the growth of axons and enhances their regeneration, treating PNS injuries	[211]
Polyacrylamide (PAM) hydrogel	Ridge/groove	Effectively regulates the growth of Schwann cells (Schwann cells derived from Sprague-Dawley rat pups (3 days old)) in anisotropic hydrogel micropatterns and increases the upregulation levels of BDNF by 40% and β-actin by 50% in Schwann cells.	[212]
Poly-L-lactic acid microfibers	Microfiber	Rostrocaudal axonal regeneration, and supraspinal neurons	[213]
PCL	Electrospun nanofibrous	Promoting adhesion, down-regulating Glial Fibrillary Acidic Protein (GFAP) expression, and enhancing neural regeneration through the reduction of astrogliosis	[214]
PCL	Electrospun nanofiber	Nerve injury repair: The orientation of dorsal root ganglia neurite growth on uniaxially aligned scaffolds	[215]
Electrospun fibers of poly(lactide-co-glycolide)	Nanofiber	Up-regulating the expression of early myelination markers promotes the maturation of Schwann cells	[216]
Gold (Au)	Nanowires	Increase in the quantity of glial cells	[217]
Gallium phosphide (GaP) (111) B epi-ready substrates	Nanowires	Enhancing the guidance of optic nerve axons and effectively promoting neurite outgrowth in retinal cells.	[218]
Silica (SiO2) nanoparticles	Nanoroughness	Role of ECM in enhancing survival: glial cell-associated changes in nanoroughness during neurodevelopment	[219]
Ultra-thin PCL/poly lactic acid	Thin films	Favorable conditions for cell attachment and proliferation, in addition to possessing outstanding mechanical properties in the films.	[220]
Poly (D, L-lactide-co-caprolactone)	Conduit/ ridges/grooves	The expedited movement of Schwann cells towards regions characterized by elevated peptide concentrations enhances the regeneration of the sciatic nerve by improving the recovery rate, restoring both functionality and microstructural integrity, diminishing fibrosis in muscular tissues, and promoting an increase in vascularization	[221]
Poly (L-lactide-co-caprolactone)	Multichannel nerve conduit	Improving Schwann cell growth in the direction of the nanofibers, along with enhancing cell growth in channels with a high density of channels	[222]

### Bioactive molecules

**Fig. 5:**
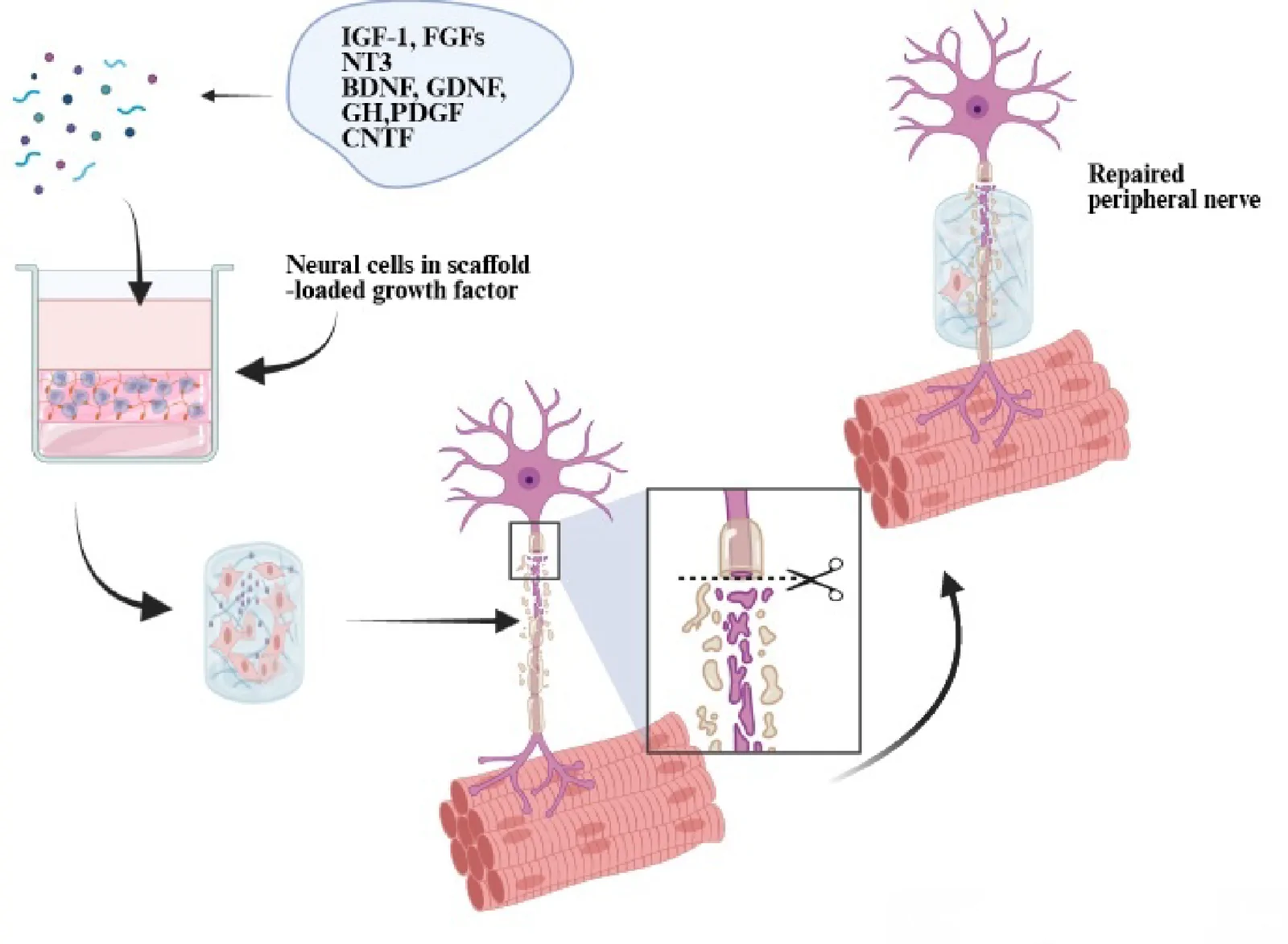
An illustrated diagram highlighting the different sources of growth factors employed in peripheral nerve regeneration. A diagram shows the process of repairing peripheral nerve damage using a cutting-edge solution that combines stem cell technology with neurotrophic factors such as NGF, IGF-1, FGF, NT3, BDNF, GDNF, GF, CNTF, and PDGF in a tissue-engineered nerve graft. After careful isolation, cultivation, and expansion, stem cells are incorporated into a neural scaffold made from neural tissue sourced from stem cells. When implanted, these stem cells differentiate, produce crucial neurotrophic factors, and actively promote the regeneration of peripheral nerves (Created with www.biorender.com)

Neural guidance channels (NGCs) can release bioactive substances embedded within biomaterials, influencing cellular behavior and enhancing cell survival and proliferation. Additionally, these biomaterials can be modified with microparticles composed of alternative materials infused with specific bioactive molecules, such as salicylic acid, to mitigate inflammation and minimize scar tissue development following peripheral nerve injuries [223]. Neurotrophic factors, including nerve growth factor (NGF) and insulin-like growth factor-1 (IGF-1), play a particularly significant role in facilitating the regeneration of peripheral nerves [224]. The brief half-life and swift degradation of growth factors within the body present significant challenges when attempting to administer them to more remote sites. The effective use of NGC relies on the careful incorporation of external growth-promoting agents into its ECM proteins to ensure sustainable localized delivery. This broad spectrum of agents includes regenerative molecules, growth factors, biochemical signals, adhesion and signaling receptors, inflammatory cytokines, bioactive compounds, and bioactive peptides, all of which have demonstrated efficacy in promoting axonal regeneration and achieving improved functional results [225, 226]. In the sector of neural tissue engineering, an impressive assortment of growth factors is routinely utilized, including neurotrophins, neurokines, and survival factors, which are classified as bioactive macromolecules. The neurotrophin collection includes significant factors such as neurotrophin-4/5 (NT4/5), neurotrophin-3 (NT-3), BDNF, and NGF. In contrast, survival factors comprise glial cell line-derived neurotrophic factor (GDNF), endothelial cell-related factors, fibroblast growth factors (FGFs), insulin-like growth factor (IGF), and ciliary neurotrophic factor (CNTF), which collectively support the survival and differentiation of neural cells, often accompanied by growth hormone (GH) or various combinations thereof. Furthermore, platelet-derived growth factors (PDGFs) serve not only as fillers for neural conduits but also as structural supports for nerve stumps [227, 228] (see Fig. 5). Neuronal growth factors are substances that play a crucial role in the development of neurons. Additionally, they contribute to tissue repair and the formation of blood vessels, rendering them intriguing candidates for promoting the integration of implants with host tissues [229].

### Conductivity properties

The NGC has the potential to showcase conductive biomaterials. Investigations into conductive polymers and electrical stimulation signify a pioneering method for augmenting neurite extension and facilitating axonal regeneration [230]. In experiments conducted on rats, electroconductive conduits were found to support sciatic nerve regeneration after eight weeks following surgery [231]. The ramifications of electrical stimulation have been thoroughly examined, revealing its effectiveness in promoting axonal regeneration and increasing catecholamine release. Additionally, electrical stimulation is recognized as a Food and Drug Administration (FDA)-approved treatment for various disorders, including Parkinson’s disease, obsessive-compulsive disorder, and depression [232, 233]. Extensive investigations have been conducted on various conductive polymers, highlighting their immense potential for developing neuronal scaffolds and electrical stimulators. Their impressive talent for delivering electrical and electromechanical signals, alongside the existence of π-conjugated electrons in unsaturated bonds, boosts their mobility within the cell and develops effective conduction pathways.Prominent examples of conductive polymers utilized in neuronal scaffolding include polypyrrole [234], polyaniline (PANi) [235], poly (3,4-ethylene dioxythiophene) (PEDOT) [236], and polythiophene [237]. The significant breakthroughs in electrical stimulation methodologies have greatly enriched our knowledge of neural networks, empowering us to activate single neurons with unparalleled accuracy. Unlike in previous decades, modern technology permits electrical stimulation with exceptional spatial accuracy [238].

### The role of stem cell and scaffold implantation in immune reactions

In tissue engineering, scaffolds act as vital foundations. The arrival of artificial medical devices triggers an array of innate immune responses, beginning with hemostasis, progressing to inflammation, and culminating in tissue regeneration. As scaffolds are introduced into the body, they interact with blood and tissue fluids, triggering the foreign body response, a crucial element of the wound-healing process. It is vital to comprehend how inflammatory cells interact with these synthetic materials. Almost every scaffold implant incites varying levels of foreign-body responses and immune reactions. It is commonly accepted that the physical and chemical characteristics are essential in shaping the tissue response to these implants. Consequently, tissue engineering scaffolds are meticulously designed with high porosity, diverse shapes and sizes, and specialized topographical and surface features to encourage cell adhesion, infiltration, and effective tissue integration across various applications. Although significant emphasis has been placed on altering scaffold properties to improve the behavior of “desired” cells, there is a glaring lack of attention to the responses of “unwanted” immune cells to specific scaffold designs. It is crucial to recognize that the body’s foreign-body response can significantly compromise the survival of transplanted cells incorporated into tissue-engineering scaffolds [239, 240].

Scaffolds frequently incorporate drug- or biomolecule-loaded micro- and nanoparticles to enhance cell migration, proliferation, and differentiation. Once released from the scaffolds, micro- and nanoparticles measuring less than 10 μm are efficiently phagocytosed by monocytes and macrophages. Conversely, scaffolds larger than this, reaching up to 100 μm, evade phagocytosis by individual monocytes or macrophages. Instead, several of these cells merge to form foreign-body giant cells, which subsequently engulf the scaffold. In larger, thicker scaffolds (> 100 μm), “frustrated phagocytosis” can occur in fused monocytes/macrophages. This reaction may result in the secretion of several cytotoxic agents, such as reactive oxygen species and proteolytic enzymes, which can compromise the integrity of the scaffold and nearby healthy tissue [241, 242].

Major endeavors in tissue engineering have focused on creating biocompatible, biodegradable implants that serve as temporary measures to promote tissue regeneration, accelerate wound healing, and enhance physiological and immune responses. Polymers such as PLLA and poly (glycolic acid) (PGA) are commonly utilized across various applications [243]. Lactic acid, the main component of these polymers, is a metabolite generated by cells, which typically leads to these degradable materials and their byproducts being viewed as having good biocompatibility. However, scaffolds derived from these substances may elicit distinct immune responses in adjacent tissues [239, 244]. Early studies reveal that as degradation occurs, material fragments and metabolites—including L-lactic acid, glycolic acid, and their oligomers—are released, leading to an accumulation that can create an acidic environment around the tissue [245]. The heterogeneous nature of stem cell therapeutics, combined with the limited experience in their development, presents a significant limitation. Additionally, the perceived risk of tumorigenicity associated with stem cell therapies remains a major concern. This concern is illustrated by a case where a tumor developed four years after fetal neural stem cell transplantation for ataxia telangiectasia [246, 247]. Investigations confirmed that the tumor originated from the transplanted material. Similar occurrences have been noted in cases involving spinal injury that utilized olfactory mucosal cell transplants; a patient complaining of back pain eight years post-treatment was ultimately found to have a mucosal-like mass at the transplant site [248]. This research is particularly relevant because the treatment employed adult stem cells, which are generally regarded as having lower tumorigenic potential than fetal or pluripotent stem cells, and in light of recent innovative spinal injury treatments involving olfactory ensheathing cells [249]. Although the authors of this study reported no adverse effects after 19 months, the possibility of tumor development from stem cell grafts many years after transplantation emphasizes the importance of thorough follow-up programs to mitigate patient risk. A considerable amount of research in tissue engineering has focused on developing undifferentiated stem cells (particularly MSCs), which are known for their immunosuppressive capabilities. The intricate mechanisms through which MSCs downregulate immune cell responses remain largely elusive. However, numerous studies have illuminated this complex interaction. Essentially, MSCs communicate with T cells via a variety of adhesion molecules, including Vascular Cell Adhesion Molecule-1, Intercellular Adhesion Molecule-1, and Lymphocyte Function-Associated Antigen-3. Their expression of major histocompatibility complexes I and II, along with Fas ligand, indicates their vital role in modulating immune responses [72–74]. MSCs are known to temporarily suppress cytotoxic T cell responses, a factor of substantial relevance in the realm of graft-versus-host disease. In in vivo research, cytotoxic T cells and Natural Killer (NK) cells trigger cytotoxic mechanisms. Additionally, by suppressing interferon-gamma, MSCs curtail NK cell activation [250–252].

## Future perspectives and conclusions

Future investigations must focus on elucidating these interactions within 3D cultures and organoids, as they provide a more accurate representation of the neural tissue microenvironment than conventional 2D cultures, which frequently constrain authentic in vivo interactions [168, 253]. Although significant advances have been made in microsurgical techniques, nerve repair still falls short of being ideal. Over recent decades, the use of nerve conduits in clinical practice has significantly enhanced functional recovery after peripheral nerve injuries, particularly when autologous grafts are not feasible. Innovative biodegradable nanomaterials with unique topographical patterns open exciting possibilities for the future of nerve conduits. When implanted with Schwann cells, these 3D scaffolds can mimic biomimetic environments found in Bunger bands, thereby encouraging axonal regrowth. Additionally, the use of NSCs within biodegradable conduits has been effective in promoting neural regeneration in clinical settings. In 2008, the Food and Drug Administration (FDA) and Conformité Européenne authorized the use of nerve conduits composed of various materials, including collagen, PGA, and PCL. Research from that time revealed that these conduits were only effective for lesions measuring under 3 cm in length in both peripheral and cranial nerves [254]. Most nerve conduits or wraps available on the market are biodegradable and are generally employed for nerve gaps that are less than 3 cm long. Research from randomized clinical trials has demonstrated that PGA conduits show efficacy comparable to that of autologous nerve grafts. In this study, researchers examined the promising capabilities of a bioabsorbable conduit (PGA) for effective nerve repair. A total of 98 participants were enrolled, leading to 136 documented cases of hand nerve transections. The subjects were categorized into two primary repair groups: the standard repair group, which consisted of end-to-end reconnections or nerve grafting, and the conduit group, which used the PGA conduit. The findings from the conduit group were remarkable: 44% achieved excellent outcomes, 30% good outcomes, and 26% poor outcomes. Notably, the extent of sensory improvement varied with the nerve gap’s length; gaps of 4 mm or less exhibited significantly better sensation following conduit repair. For nerve defects of 8 mm or more, reconstruction was accomplished using either nerve grafts or conduits. This study shows that conduit repair significantly improves sensory outcomes in nerve gaps measuring 4 mm or less compared to complete digital nerve repairs. Furthermore, PGA conduit repair is superior to nerve grafting for larger nerve gaps, while minimizing donor-site complications associated with graft harvesting [255]. The findings further highlight that PGA conduits, along with autologous vein conduits and polylactide-caprolactone (PLCL) conduits, achieve similar effectiveness as autologous nerve grafts. The purpose of this study was to explore improvements in sensory nerve function following treatment of traumatic peripheral nerve injuries with a biodegradable PLCL Neurolac nerve guide, compared with standard reconstructive approaches. In total, 30 patients participated in this randomized, multicenter study, which documented 34 nerve injuries. Following a brief learning period, implanting the nerve guide proved quite straightforward. While the experimental group reported more complications, none were directly linked to the device. The sensory improvements noted in the nerve guide group were at least on par with those observed in the control group. In conclusion, this research determined that the Neurolac nerve guide represents an effective solution for repairing severed peripheral nerves in the hand [256]. Additionally, collagen conduits have similar effectiveness compared to direct microsurgical neurorrhaphy [257, 258].

Currently, the availability of clinically effective products designed to facilitate neural regeneration is primarily limited to cases involving peripheral nerve injuries. Recent developments in materials science and tissue engineering have introduced non-resorbable, inert polymers, including silicone and expanded polytetrafluoroethylene (PTFE), along with biodegradable synthetic polymers such as PGA and PLCL. Evidence suggests nerve regeneration outcomes are more favorable with silicone tubes than PTFE. Nevertheless, numerous patients have reported complications, including graft extrusions, sensations of foreign bodies, and instances of nerve compression, which have occasionally required the surgical removal of silicone implants [259]. Our research highlights the crucial role of Schwann cells in axonal regeneration and myelination, which is associated with enhanced long-term functional outcomes. Given their remarkable versatility, we strongly support increased focus on Schwann cells in future research and clinical efforts aimed at neural regeneration [260]. The success of transplanted cells heavily relies on a well-structured microenvironment, which must incorporate optimal contact guidance, biochemical signals, topographical features on biomaterials, and directional inputs via conduits. This setup is expected to enhance the functionality of both implanted neural stem cells and local Schwann cells, thereby promoting more effective neural regeneration. In addition, cutting-edge conduit designs can be made possible using techniques such as stereolithography or bioprinting, facilitating the quick development of sophisticated and accurate nerve guidance structures that can be customized for each patient. Additionally, the interactions between cells and biomaterials will likely affect cell behavior and directional movement, presenting a promising area for further exploration and research. Integrating material science, cellular biology, and engineering methodologies can enhance functional recovery following nerve injuries. The drawbacks associated with non-resorbable implants have resulted in a growing inclination towards biodegradable NGCs. In specific scenarios involving the repair of the CNS and PNS, biomaterials must exhibit characteristics that closely resemble those of native brain tissue, spinal cord, and peripheral nerves. The challenge of CNS repair necessitates that the dimensional properties of these biomaterials be meticulously adjusted to align with the mechanical and biochemical properties required for effective integration. Furthermore, given the inherent complexity of the CNS, it is essential to incorporate a variety of cell types, including oligodendrocyte progenitor cells, astrocytes, and microglia. Current research suggests that greater emphasis should be placed on technological innovations at the topological level to facilitate axonal alignment and enhance functionality in the context of peripheral nerve injury repair. Additionally, addressing the challenge of repairing long-distance lesions (> 10 mm) remains critical [261]. Thus, integrating various cell types and modification strategies presents promising techniques for advancing PNS repair. Integrating topographic design with specific materials can yield contact signals, such as molecules derived from the ECM and nerve growth factors, essential for promoting nerve regeneration. Current research in this area is advancing rapidly. The synergy of topographic design, guidance stimuli through scaffolding, and sophisticated biological and engineering techniques addresses unmet clinical needs, particularly for patients suffering from CNS and PNS injuries. The urgent global health challenges we face today, coupled with the need to repair damaged tissues, have sparked a wave of innovative strategies, designs, and technologies focused on restoring functional tissues. In the field of neural tissue engineering, we face notable challenges that require resolution: firstly, grasping the complex interactions between cells and nanostructures, which are essential in influencing cell fate, phenotype, and behavior; secondly, fabricating materials that comply with strict biocompatibility standards, including safety, synthesis, bio-interactions, topography, controlled release, and electrical conductance, all while upholding rigorous quality control and consistent reproducibility. As this advancing field develops, scaffolds are emerging as an innovative solution that offers a multitude of benefits. By adopting this methodology, we are laying the groundwork for the creation of scalable medical devices that effectively emulate the natural ECM environment. Every step forward in scaffold engineering extends the possibilities and brings us nearer to a future in which injured individuals can recover and find hope.

## Data Availability

No datasets were generated or analysed during the current study. Data sharing is not applicable to this article, as no datasets were generated or analyzed during the current study.

## Abbreviations

ECM: Extracellular matrix
ND: Neurological disorder
CNS: Central nervous system
PNS: Peripheral nervous system
NSCs: Neural stem cells
ESCs: Embryonic stem cells
MSCs: Mesenchymal stem cells
iPS: induced Pluripotent stem cell
2D: 2-dimensional
SAPs: Self-assembling peptides
PCL: Polycaprolactone
PPSu: Polyphenylene sulfone
BMP-4: Bone morphogenetic protein 4
BDNF: Brain-derived neurotrophic factor
RGD: Arginine-glycine-aspartic acid
PDGF: Platelet-derived growth factor
IL-6: Interleukin-6
IKVAV: Isoleucine-lysine-valine-alanine-valine
NCSCs: Neural crest stem cells
BSCB: Blood-spinal cord barrier
HA: Hydroxyapatite
Ms: Magnetization
SDF-1: Stromal cell-derived factor 1
3D-GF: 3D graphene foam
sEVs: Small extracellular vesicles
SCI: Spinal cord injury
PLGA: Poly (D, L-lactide-co-glycolide) acid
bFGF: Fibroblast growth factor
DRGs: Dorsal root ganglia
NPCs: Neural precursor stem cells
Rap1: Ras-associated protein-1
FOXO: Forkhead box O
Ang1: Angiopoietin-1
NgR-Ab: Antibody to the Nogo receptor
TBI: Traumatic brain injury
NPCs: Neural precursor cells
SGNs: Spiral ganglion neurons
HS: Heparan sulfate
PLLA: Poly-L-lactic acid
MAPK: Mitogen-activated protein kinase
PI3K: Phosphatidylinositol 3-kinase
AIS: Association Impairment Scale
mRS: Modified Rankin Scale
RP: Rapid prototyping
UV: Ultraviolet
AI: Artificial Intelligence
ML: Machine learning
DL: Deep learning
FFNN: Feedforward neural networks
BNN: Bagged neural networks
PSO: Particle swarm optimization
SWIRL: Swarm intelligence reinforcement learning
BBB: Blood-brain barrier
hESCs: Human embryonic stem cells
YIGSR: Tyrosine-isoleucine-glycine-serine-arginine
PUA: Polyurethane acrylate
EVA: Ethylene-vinyl acetate
PAM: Polyacrylamide
GFAP: Glial Fibrillary Acidic Protein
Au: Gold
GaP: Gallium phosphide
NGCs: Neural guidance channels
NGF: Nerve growth factor
IGF-: Insulin-like growth factor-1
NT4/5: Neurotrophin-4/5
NT-3: Neurotrophin-3
FGFs: Fibroblast growth factors
CNTF: Ciliary neurotrophic factor
GH: Growth hormone
FDA: Food and Drug Administration
PANi: Polyaniline
PEDOT: Poly (3,4-ethylene dioxythiophene)
PPy: Polypyrrole
CNTs: Carbon nanotubes
PGA: Poly (glycolic acid)
NK: Natural Killer
PLCL: Polylactide-caprolactone
PTFE: Polytetrafluoroethylene
